# 15th Anniversary of *Genes*: Advances and Perspectives in Neurogenetics and Neurogenomics

**DOI:** 10.3390/genes17101237

**Published:** 2026-10-08

**Authors:** Claudia Ricci

**Affiliations:** Department of Medical, Surgical and Neurological Sciences, University of Siena, 53100 Siena, Italy; claudia.ricci@unisi.it

The 15th anniversary of *Genes*, alongside the 10th anniversary of its initial Impact Factor, marks a significant milestone in reflecting on the remarkable advancements in the field of human molecular genetics. Over the past 15 years, neurogenetics and neurogenomics have undergone a transformative shift. The field has advanced from identifying single-gene monogenic mutations using targeted technologies to adopting high-throughput multi-omics integration, comprehensive system-level bioinformatic modeling, and AI-powered precision medicine.

Neurodevelopmental, neurovascular and neurodegenerative disorders present significant clinical and socioeconomic challenges in contemporary medicine. Understanding their intricate biological origins requires a variety of approaches, from detailed functional analysis of specific genomic regions to extensive, system-wide genomic studies. This Special Issue gathers key contributions that highlight the evolution of this scientific field. The papers cover topics including monogenic pathogenetic mechanisms, structural copy number variants, and associations at the epigenome and transcriptome levels. They also discuss cutting-edge multi-omics computational platforms and progressive frameworks for studying neurodegenerative diseases.

Some of the papers in this Special Issue focus on the level of individual genes, where specific variants cause protein modifications that lead to structural instability and functional alteration, resulting in disease or predisposition to pathological conditions. In this context, Figura et al. [1] investigated Triosephosphate Isomerase 1 (TPI1) deficiency, a rare and devastating autosomal recessive glycolytic enzymopathy characterized by hemolytic anemia, neuromuscular dysfunction, and early lethality. The authors performed detailed biochemical and functional analyses on the pathogenic *TPI1^R5G^* substitution, a novel variant identified in a heterozygous compound patient carrying a frameshift mutation in one allele and the R5G substitution in the other. This variant results in atypical TPI deficiency, which lacks anemia and has more slowly developing neurological and locomotor impairment. The authors demonstrated that the TPI^R5G^ protein exhibited wild-type activity and modestly increased dimer stability. Nevertheless, there was a significant decrease in the steady-state levels of TPI protein. This suggests that the instability of the mutant protein is the underlying cause of the disease. Furthermore, the study revealed that three recently identified compounds, which were screened for novel TPI deficiency therapies, significantly increased TPI protein levels in patient cells. The rise in TPI protein levels caused a significant increase in TPI activity since the mutant protein essentially maintains wild-type activity. This offers vital proof-of-concept insights for potential therapies in patients with TPI deficiency.

Amico et al. [2] expanded the clinical scope of single-gene variants by evaluating the impact of heterozygous loss-of-function variants in *ABCC6*, an ATP-binding cassette transporter gene. Biallelic causative variants in this gene are associated with generalized arterial calcification of infancy (GACI) and pseudoxanthoma elasticum (PXE). By analyzing a cohort of patients presenting with premature ischemic stroke and cerebrovascular disease, the authors revealed that *ABCC6* heterozygosity—previously considered clinically silent—serves as an independent genetic predisposing factor for accelerated arterial calcification and early-onset cerebrovascular events. This work highlights the importance of re-evaluating carrier status in autosomal recessive genes as potential modifiers of complex vascular phenotypes. This has the potential to refine risk stratification and inform management strategies for patients with conventional vascular risk factors.

Focusing on monogenic alterations responsible for developmental structural disorders, Silvey et al. [3] examined the *POLR1A* gene, which encodes the largest catalytic subunit of RNA polymerase I. Pathogenic variants in the *POLR1A* gene are classically associated with craniofacial and developmental syndromes, including Acrofacial Dysostosis-Cincinnati, which exhibits features similar to those of Sweeney–Cox, Saethre–Chotzen, Treacher–Collins types 2–4, and Robinow–Sorauf syndromes. The authors’ objective was to shed light on the shared pathogenic mechanisms underpinning these conditions, proposing that they fall within the spectrum of RNA polymerase I (Pol I)-related ribosomopathies. They performed a detailed clinical evaluation of a patient with a heterozygous *POLR1A* variant (p.Met496Ile). To assess the impact of mutations, protein-protein and gene-gene interactions were analyzed using STRING and Pathway Commons. The patient’s features overlap with those of the Sweeney–Cox, Saethre–Chotzen, Robinow–Sorauf and Treacher–Collins types 2–4, suggesting a continuum. Computational analyses revealed the partners and pathways associated with POLR1A, which are involved in Pol I function, nucleolar processes, and ribosomal biogenesis. Structural modeling of the Met496Ile variant showed that it disrupts DNA binding and polymerase activity. This establishes a link between molecular dysfunction and clinical phenotypes. The results indicate that several neurodevelopmental disorders may in fact be components of a developing spectrum of disorders related to Pol I genes, suggesting that they are not entirely distinct entities. Acknowledging these links might have consequences for genetic testing methods, counseling, and medical management in the future.

Neurodevelopmental disorders are also the subject of two studies focused on structural chromosomal alterations and the complex genomic landscape. La Monica et al. [4] conducted a comprehensive genetic and clinical characterization of copy number variations (CNVs) on chromosome 16p within a large Italian pediatric cohort presenting with Autism Spectrum Disorder (ASD), Intellectual Disability (ID), and Developmental Delay (DD). The study focused on microdeletions and microduplications involving 16p11.2, 16p13.3, and 16p13.11. By integrating high-resolution chromosomal microarray analysis with detailed deep phenotyping, the authors identified distinct genotype–phenotype correlations. Specifically, deletions in the 16p region were primarily associated with developmental delay, language impairments, and ASD-related characteristics, while duplications were more often connected to behavioral dysregulation, attention deficits, and varying degrees of cognitive impairment. Marked phenotypic variability was observed among individuals with similar CNVs, which suggests that modifying genetic or environmental factors also play a role. In a subset of patients, additional CNVs were identified, which potentially made the clinical severity worse, in line with the two-hit model. These results highlight the value of systematic genomic screening for diagnosis and the importance of an integrated genotype–phenotype approach to enhance clinical interpretation, management and genetic counseling in NDDs.

Complementing this structural perspective, Payne et al. [5] examined the Phelan–McDermid Syndrome (PMS), an NDD caused by deletions in the 22q13.3 region or pathogenic/likely pathogenic variants in the SH3 and multiple ankyrin repeat domains 3 (*SHANK3*) gene. PMS can present with developmental delay, moderate to severe intellectual disability, absent or severely delayed speech, neonatal hypotonia, autism spectrum disorder (ASD), and other problematic behaviors. In light of the significant clinical heterogeneity among PMS individuals, the authors combined multi-domain behavioral testing with genetic characterization and plasma metabolomic profiling. Their integrated analyses revealed that individuals with *SHANK3* variants exhibited lower adaptive behavioral skills than those with 22q13 deletions, irrespective of deletion size. Conversely, genomic parameters were mostly unrelated to Autism Diagnostic Interview-Revised (ADI-R) scores. Metabolic profiling revealed distinct profiles in individuals with PMS compared to the control group. Furthermore, it distinguished between those who met or did not meet the ADI-R ASD cutoff. Cluster analyses identified groups of patients with ASD and other clinical characteristics. These findings emphasize the value of combining genetic profiling with systemic metabolomics to identify functional biomarkers and improve patient classification in neurodevelopmental disorders.

Dalsberg et al. [6] adopted a high-throughput systems genomics approach to studying neurodevelopmental disorders, providing a novel epigenetic and transcriptional framework for Tic Spectrum Disorders (TSDs), including Tourette syndrome. Although heritable components have been linked to TSD, no clear genetic mechanisms have been found yet. There are still important questions about what causes TSD, especially about how the environment might play a role in its development. To dissect environmental and post-zygotic factors from genetic confounding, the authors performed an RNA sequencing and DNA methylation analysis on monozygotic twin pairs, either discordant or concordant for TSD severity. They identified a dozen genes that were differentially expressed between individuals with and without TSD, most of which were long non-coding RNAs or pseudogenes. The expression of the small RNA gene *RNY1* was significantly associated with tic severity, which suggests the involvement of immune-related processes. Expression quantitative trait methylation (eQTM) analysis identified 236 genes associated with methylation. Gene set enrichment analysis revealed widespread downregulation of pathways related to translation, RNA processing and neurobiological functions in TSD individuals. This elegant twin study suggests that environmentally influenced gene expression may contribute to TSD pathogenesis by dysregulating immune and neuronal pathways and shows how epigenetic modifications act as a crucial mechanistic link between early neurodevelopmental vulnerability and phenotypic expression.

With the exponential growth in the complexity of neurogenomics data, innovative bioinformatic and whole-genome approaches are essential for discovering novel mechanisms and diagnostic markers. In their study of high-dimensional transcriptional dynamics, Kobayashi and Taguchi [7] employed a fully data-driven approach involving unsupervised feature extraction based on tensor decomposition (TD) [8] to analyze somatostatin (Sst)-expressing interneuron transcriptional states during sleep–wake cycles. Applying this approach to publicly available gene expression profiles from the male mouse cortex under sleep-deprivation (SD) conditions [9] revealed subtle, nonlinear gene expression programs that were regulated during non-REM and REM sleep phases and which had been missed by traditional linear differential expression analyses. They identified two different patterns, “altered during SD but not recovered during RS” and “altered during SD with an overshoot during recovery sleep (RS)”, which differ from the commonly expected pattern “altered during SD and recovered during RS”. Enrichment analysis of the genes associated with these patterns revealed enrichment in Sst-expressing cell types. These results were confirmed by uploading both gene sets to AlphaGenome and retrieving predicted DNase-Seq data. The top-ranked genes that distinguish various brain subregions from the whole brain were enriched in Sst cell types. These findings suggest that Sst-expressing neurons play a crucial role in sleep deprivation (SD) and offer new insights into the transcriptomic homeostasis of GABAergic cortical networks during sleep. This validates TD-based algorithms as effective tools for analyzing complex neurogenomic datasets.

In the field of clinical genomic diagnostics, Then et al. [10] demonstrated the effectiveness of whole-genome sequencing (WGS) in resolving previously unexplained neurodevelopmental and neurodegenerative phenotypes. In this instance, the analysis was conducted to determine the molecular genetic basis of a disorder affecting Petit Bleu de Gascogne dogs, which was characterized by hind-limb weakness, anxiety, hallucinatory episodes, lip smacking, progressive vision loss, muscle atrophy and brain ataxia. WGS successfully identified novel biallelic missense variants in the *AP3B1* and *TRAPPC9* genes, which encode proteins implicated in sorting and transporting proteins to lysosomes via the Golgi apparatus. Detailed microscopic examinations were conducted to define the disease pathology. These studies revealed significant accumulations of autofluorescent intracellular inclusions in the brain, retina and heart. These inclusions exhibited ultrastructural properties comparable to those of lysosomal storage bodies found in neuronal ceroid lipofuscinoses (NCLs), a group of inherited, progressive, lysosomal storage-related neurodegenerative disorders. This information implies that the disease may be the result of a combination of the two variants whose encoded proteins interact in the Golgi apparatus, facilitating the sorting and transport of proteins to lysosomes. The study emphasizes the importance of WGS in overcoming the limitations of targeted gene panels in terms of coverage, enabling the identification of mutations in multiple genes for precision diagnosis.

The final two papers in this Special Issue focus on a key challenge for the future of neurogenetics and neurogenomics. With global populations aging rapidly, neurodegenerative diseases such as Alzheimer’s and Parkinson’s diseases represent an unprecedented public health crisis. Beyond their devastating biological impact on brain architecture, these progressive disorders impose an immense socioeconomic burden on healthcare systems worldwide. They account for hundreds of billions of dollars in direct medical costs and long-term care infrastructure every year. Furthermore, the non-monetary consequences, characterized by severe caregiver burnout, emotional distress, loss of independence, and family disruption, highlight the urgent need for early diagnostic tools and disease-modifying therapies that can delay the onset of symptoms or slow their progression.

In their analysis of population-scale genomic data from the All of Us Research Program, Abe and Niemchick [11] investigated the risk of Parkinson’s disease (PD). Using the All of Us Dataset, which contains genomic, blood test, and other environmental data, the authors explored the interplay between genetic dysfunction and environmental factors by obtaining odds ratios (ORs) and adjusted ORs using multivariable logistic regression. The research focused on genetic susceptibility loci on chromosome 12, which includes the leucine-rich repeat kinase 2 (*LRRK2*) gene. *LRRK2* missense mutations, particularly the G2019S mutation, are the most common cause of monogenic PD, but they are relatively rare in the overall PD population. GWAS of PD have identified genetic variants in and near the *LRRK2* gene as PD risk factors in individuals with no other known genetic causes of PD [12]. For this reason, the authors investigated the relationship between genetic polymorphisms in the *LRRK2* gene and other loci on chromosome 12 and PD. They identified thousands of polymorphisms; of these, fourteen had high ORs, which were similar to those of the *LRRK2* gene G2019S mutation. Five of these were significantly altered when demographic and environmental factors were taken into account, particularly calcium, vitamin D and alcohol intake. The results suggest that the progression of some PD cases caused by certain polymorphisms can be delayed or prevented by environmental factors. In the future, studying the association of other factors with PD, including other chromosomes and increasing the number of participants, could improve our understanding of the interaction between genetic and environmental factors, thereby improving the design and precision of future clinical trials.

Finally, D’Argenio et al. [13] presented a landmark review entitled ‘Alzheimer’s 2030: From Precision Genomics to Artificial Intelligence’. In it, the authors synthesize the current state and future trajectory of Alzheimer’s disease (AD) research, tracing the transition from early single-marker studies, such as APOE genotyping, to more recent polygenic risk scores (PRS), single-cell PRS and fluid multi-omic biomarkers. The authors examine the influence of genetic and gender-related factors by analyzing large-scale genome-wide association studies (GWAS) and investigating their role in developing advanced genetic risk scores (GRS) for precision genomics. Despite their growing methodological sophistication, current PRS models for Alzheimer’s disease (AD) still have significant limitations that affect their clinical use. The predictive performance of PRS is only modest at the individual level, even when they are combined with age and APOE genotype. For this reason, the authors extended their analysis to include sex, gender, and sociocultural determinants in AD. Given the complexity of the data, a key focus of the review is the transformative role of artificial intelligence (AI) and machine learning (ML) in AD. The review explores the application and potential of AI in managing modifiable dementia risk factors, assessing personal risk, promoting healthier lifestyles, tailoring interventions and monitoring disease progression over time. The authors conclude with a perspective on a limited number of concrete objectives over the next five years. Firstly, large-scale, multi-ancestry genomic studies incorporating sex-stratified analyses are needed to improve PRS models and enable equitable genetic risk prediction. Secondly, there is a need to integrate sex- and gender-informed biomarkers to improve diagnosis, risk stratification, and treatment monitoring. Thirdly, developing and validating AI models that are designed to incorporate sex- and gender-related exposures and ancestry is essential to ensure clinical reliability and fairness. These models must be transparent and capable of auditing bias. Finally, longitudinal and pragmatic studies are needed to evaluate the potential of AI-personalized lifestyle interventions to modify AD risk trajectories across different populations.

In conclusion, the articles in this 15th Anniversary Special Issue demonstrate the significant advancements in neurogenetics and neurogenomics over the past fifteen years. Topics covered include resolving single-gene metabolic defects and structural copy number variants, utilizing twin epigenomics and tensor-based transcriptomics, and investigating the interactions between genetic and environmental factors and AI-driven frameworks. Together, these contributions demonstrate how the field has evolved into a comprehensive, data-driven science. Looking ahead to the next decade, the convergence of whole-genome sequencing, multi-omics integration and artificial intelligence offers the potential to alleviate the growing global impact of neurological disorders by speeding up the process of translating genomic discoveries into personalized, highly precise therapeutic interventions.

## Data Availability

No new data were created or analyzed in this study. Data sharing is not applicable to this article.
